# Factors influencing the derivation and clinical application of blood calcium adjustment equations

**DOI:** 10.1177/00045632221131673

**Published:** 2022-10-14

**Authors:** Georgia Conrich-Wilks, Fiona Ivison, Eric S Kilpatrick

**Affiliations:** 1 Department of Medical Sciences, University of Manchester, Manchester, UK; 2 Department of Clinical Biochemistry, Manchester Royal Infirmary, 5293 Manchester University NHS Foundation Trust, Manchester, UK

**Keywords:** Adjusted calcium, calcium status, calcium metabolism

## Abstract

**Background:**

Laboratories are recommended to use patient data to derive their local adjusted calcium (adjCa) equation, using numerous criteria to exclude patients with potential calcium metabolism abnormalities. It is not known which, if any, of the exclusions influence the final equation formula, or to what extent. This study investigated the effect using fewer exclusions has on adjCa equations and on patient results when compared to a reference equation.

**Methods:**

A reference ACB adjCa equation was derived from the total calcium and albumin pairs of 1305 individuals who, from an initial 22,906 adults, met recommended criteria (excluding abnormalities in either calcium, albumin, creatinine, magnesium, ALP or ALT, and specific clinical areas). This reference equation was compared to seven alternatives derived using fewer criteria, including one with no exclusions. All equations were applied to a validation cohort (*n*=19,640) to determine their effect on adjCa results and on categorizing patients into hypo-, normo- or hypercalcaemia.

**Results:**

Most alternative adjCa equations, including the one without any exclusions, showed no statistical (*p* < 0.05) difference in their slope or intercept compared to the ACB reference. Nor did any of the validation cohort have a clinically significantly different adjCa result (>5% and >0.1 mmol/L different) when applying an alternative rather than the reference equation. Additionally, no alternative equation changed the kappa categorization of the validation population’s calcium status.

**Conclusions:**

When deriving adjCa equations, most exclusion criteria have little influence on the equation or patient results, including using none at all. This knowledge could simplify deployment of local equations.

## Introduction

Only about 50% of the total serum calcium circulating in the blood is in its biologically active free or ‘ionized’ form.^1,2^ Of the rest, 35–40% of circulating calcium is bound to albumin and globulins while another 10–15% circulates bound to anions such as bicarbonate, phosphate, citrate and lactate.^1^ Despite generally being considered a more accurate indicator of true calcium status compared to total calcium concentrations, concerns about the stringent pre-analytical requirements of measuring ionized calcium restricts its use in routine practice.^1,3,4^ However, total calcium concentrations only provide an estimate of the biologically active portion of calcium based on the relationship between calcium and its binding partners.^5^ Any changes to factors influencing this relationship, such as pH and albumin concentration, can affect total calcium measurements.^5^ UK clinical laboratories therefore routinely adjust total calcium concentrations for albumin, the predominant plasma protein in the circulation, to mitigate for variation in albumin concentration.^5^

Since the 1970s, numerous calcium adjustment equations have been derived based on the linear regression between total calcium and albumin.^6^ This is generally performed in a population of patients as free from calcium homoeostasis disorders as possible since these disorders are assumed to affect the relationship, and thus the regression, between calcium and albumin.^7^ Traditionally, the most frequently used calcium adjustment equation, sometimes named Payne’s equation, involves increasing the total calcium concentration by 0.02 mmol/L for every 1 g/L the albumin concentration is below 40 g/L and vice versa, and it is often still in use today.^8^ However, more recent evidence suggests that the use of a singular calcium adjustment equation, such as Payne’s and other literature-derived equations, may not be applicable across all laboratories and patient cohorts.^9–12^ In fact, many literature-derived equations have been found to significantly misclassify patient calcium status when compared to ionized calcium, sometimes even more poorly than unadjusted total calcium concentrations.^9–12^ This has largely been attributed to differences in both the assay and analytical platform used to measure calcium and albumin which can affect the performance of the resulting calcium adjustment equation when used across different laboratories.^6,13^

To improve the validity of calcium adjustment, it has been suggested that laboratories derive equations locally from a subset of their own patient populations, with the intention to exclude those whose other biochemistry tests may indicate a potential calcium homoeostasis disorder.^6^ For example, those patients with abnormally high alkaline phosphatase or serum creatinine would be excluded. In an attempt to further harmonise laboratories’ approach to deriving equations, the Association for Clinical Biochemistry and Laboratory Medicine (ACB) published their own dataset exclusion criteria.^8^ Although largely based around the exclusion criteria originally used by Barth and Payne, some modifications and important additions were made which require more specialist tests, such as vitamin D and PTH, to have been taken concurrently.^6,8^ Consequently, local derivation of adjustment equations can be complicated in practice by the paucity of retrospective patient data that meet all the stated criteria. It is, therefore, unsurprising that there remain inconsistencies between laboratories in the exclusion criteria used when deriving adjustment equations.^5,14–17^ Furthermore, the ongoing research into whether age or population-specific adjustment equations could increase the validity of calcium adjustment in specific patient groups may be hindered by these small derivation groups.^14^

Although empirically understandable, little evidence was and is available for the exclusion criteria chosen by Barth and Payne that have subsequently been used by the ACB.^6,8^ It is therefore not known if, or by how much, each of these criteria influence the final equation, and therefore whether differences in how laboratories calculate adjusted calcium might lead to clinically different results.^6^ In this study, we aimed to identify the major exclusion factors which influence the derivation of a calcium adjustment equation by comparing the effect of different ACB exclusion criteria on the resulting adjustment equation and subsequent adjusted calcium value.

## Methods

### Data Collection

Anonymised retrospective data was obtained from the laboratory database of the Oxford Road Campus and Trafford sites of Manchester University NHS Foundation Trust (MFT). This included all measurements of total (serum) calcium, albumin, ALP (all included in a bone profile), ALT, magnesium and creatinine over a 4-month period (March to June 2021) and was collected from multiple clinical subpopulations, including from inpatients, outpatients and primary care. Test results were deemed to have been concurrent if they were collected on the same day. Measurements were divided into two 2-month periods; measurements from March and April were used for equation derivation and, for validation, the resulting equations were applied to data from May and June. Where multiple calcium and albumin measurements were made on the same patient during the 2-month period then the set with the most relevant additional tests was used and where there were multiple instances of equal additional test numbers on the same patient then the earliest set for each patient was included in the analysis.

### Laboratory Analysis

Total calcium, albumin, ALP, ALT, magnesium and creatinine were all measured on a Roche Cobas c system in all laboratories (Roche Diagnostics Limited, Burgess Hill, West Sussex, UK). Total calcium was measured using a 5-nitro-5-methyl-BAPTA (NM-BAPTA) spectrophotometric method. Albumin was measured using the Roche bromocresol (BCP) method. During the data collection period the within-lab analytical CV for total calcium was 1.6% at 1.54 mmol/L and 1.3% at 3.28 mmol/L, while for albumin this was 2.3% at 23.7 g/L and 2.0% at 38.6 g/L.

### Equation Derivation

Adjusted calcium equations were derived from data collected over a 2-month period using the protocol outlined in the ACB guidelines.^8^ Patients were only part of the equation derivation group if they met the relevant criteria (see below). Least squares linear regression of total calcium against albumin was performed, and the intercept and slope values were obtained from each derivation group. The intercept and slope values obtained were inputted into the standard equation below:

Adjusted calcium (mmol/L) = total calcium (mmol/L) – (slope * albumin (g/L)) + (2.4 – intercept)

The value 2.4 was obtained by finding the mean of the laboratory reference interval for total calcium (2.2–2.6 mmol/L) for all ages. The resulting equation can be rearranged to:

Adjusted calcium (mmol/L) = total calcium (mmol/L) – slope * (albumin (g/L) – constant)

The equation constant can therefore be found by subtracting the intercept value from the midpoint of the reference range and then dividing the number by the slope value (equation constant = (2.4 – intercept)/slope).

### Reference ACB test exclusion criteria

Patients were excluded from the calcium adjustment equation analysis according to a set of biochemical and patient exclusion criteria, based on the ACB guidelines.^8^ They were thus excluded if they met one or more of the following criteria: under the age of 18 years; attending the departments of Endocrinology, Nephrology or Haematology; total calcium < 2 or >2.7 mmol/L; albumin < 20 or >50 g/L; ALP > upper age-specific reference limit; ALT > sex-specific upper reference limit; magnesium < 0.7 mmol/L; and creatinine > 200 μmol/L. The ACB guidelines also recommend exclusion of patients based on PTH and vitamin D measurements, but we were not able to include these criteria due to an insufficient number of requests for these tests. Furthermore, patients with malignancy and patients on parenteral nutrition were not easily identifiable on the hospital laboratory information system and therefore did not form part of the exclusion criteria for our analysis. Nonetheless, for comparison purposes, we have referred to this as the ‘reference’ ACB group.

### Comparison of the test exclusion criteria which influence the adjusted calcium equation

Different subsets of ACB exclusion criteria were used to create seven further equation derivation groups of varying sizes which differed by not requiring one or more of the reference ACB’s biochemical test criteria (Table 1). The adjusted calcium equations derived from these seven groups were compared to the reference ACB equation in several ways.

**Table 1.** Reference ACB criteria derivation group characteristics (*n* = 1305).

Sex	581 (45%) female, 723 (55%) male, 1 (<1%) unknown
Age	53.8 (18.0–98.2) years, median (range)
Total calcium	2.31 (0.12) mmol/L, mean (SD)
Albumin	35.8 (5.5) g/L, mean (SD)

Firstly, the slope and intercept values were compared to the reference ACB criteria equation using z-values to establish if there was any statistical difference in either or both line components. Z-scores were calculated for intercept values as following: (ACB intercept value – exclusion criteria intercept value) /√ ([standard deviation of ACB intercept value]^2^ + [standard deviation of exclusion criteria intercept value]^2^). The same was repeated with slope values.

Secondly, the eight adjustment equations were then applied to calcium and albumin results collected from adults during the 2-month validation period. The prevalence of any clinically significant differences between the adjusted calcium concentration derived from the ACB equation and that derived from each of the other seven groups was established in all adults. A clinically significant difference was defined as being over 5% or 0.1 mmol/L different (whichever was greater) from one another, as recommended by Jassam et al.^14^

Thirdly, patient calcium status was also classified according to Pathology Harmony reference ranges (adult: 2.2–2.6 mmol/L) into hypo-, normo- or hypercalcaemic categories for all eight adjustment equations.^8,18^

Finally, the unadjusted mean total calcium and albumin concentrations of the ACB criteria group were compared with the other seven groups using two-tailed t-testing, assuming unequal variances because of the large differences in group patient numbers.

### Statistical Analysis

All statistical analyses and descriptive statistics were performed by Analysis Toolpak and Real Statistics Excel packages (Microsoft Excel 2019) (version 16.52). A *p*-value < 0.05 was considered statistically significant across all statistical analyses. Cohen’s kappa statistic was used to measure how closely, or otherwise, the adjusted calcium concentrations derived from different adjustment equations agreed with the reference ACB equation on classifying the calcium status of the validation group. Unadjusted calcium was also compared with the ACB reference using the same statistic.

## Results

Overall, 50,671 pairs of total calcium and albumin measurements were collected over March and April 2021. When multiple paired results from the same patient were removed, 26,316 pairs remained (22,906 adult patients, 3410 paediatric patients). Of the 22,906 adults (12,289 female, 10,614 male, three unknown; median age: 55.7 years [range 18.0–101.4]), 1305 met the ACB test exclusion criteria as described in the Methods section and formed the reference equation derivation group (Table 1).

### Adjustment equations and their comparison

Calcium adjustment equations derived from groups where only a subset of the ACB exclusion criteria were applied were statistically compared to the reference ACB equation (Table 2). Most of the subset groups’ slope and intercept values did not statistically significantly differ from those of the reference ACB group. Perhaps surprisingly, the derivation group with the statistically closest intercept and slope values compared to that of the reference ACB was the group with no exclusions (Group 8). This derivation group was also the only one to result in a numerically higher slope value than the reference ACB equation and the only group to include extreme total calcium (<2.2 and >2.7 mmol/L) and albumin (<20 and >50 g/L) concentrations. When these extreme values were removed to give the calcium and albumin criteria group (Group 7), the slope value was markedly reduced. Indeed, Group 7 produced the most significantly different slope and intercept value compared to the reference ACB group.

**Table 2.** The effect of different exclusion criteria on the linear regression of total calcium against albumin. *p < 0.05, **p < 0.01.

Derivation group	Exclusion criteria	Derivation group size (*n*)	Slope		Intercept	
			Value (95% CI)	*p*-value	Value (95% CI)	*p*-value
1. All ACB exclusion criteria	Total calcium (<2.0 or >2.7 mmol/L), albumin (<20 or >50 g/L), ALP (>URL), Mg (<0.7 mmol/L), ALT (>URL), creatinine (>200 μmol/L), departmental exclusions	1305	0.0148 (0.0139–0.0158)		1.780 (1.746–1.815)	
2. All ACB exclusion criteria bar Mg	Total calcium (<2.0 or >2.7 mmol/L), albumin (<20 or >50 g/L), ALP (>URL), ALT (>URL), creatinine (>200 μmol/L), departmental exclusions	13,020	0.0141 (0.0137–0.0144)	0.159	1.818 (1.805–1.830)	0.046
3. All ACB exclusion criteria bar creatinine	Total calcium (<2.0 or >2.7 mmol/L), albumin (<20 or >50 g/L), ALP (>URL), ALT (>URL), departmental exclusions	1755	0.0144 (0.0136–0.0152)	0.503	1.795 (1.768–1.823)	0.497
4. All ACB exclusion criteria bar ALT	Total calcium (<2.0 or >2.7 mmol/L), albumin (<20 or >50 g/L), ALP (>URL), creatinine (>200 μmol/L), departmental exclusions	2518	0.0144 (0.0137–0.0151)	0.497	1.793 (1.770–1.816)	0.549
5. All ACB exclusion criteria bar both creat and ALT	Total calcium (<2.0 or >2.7 mmol/L), albumin (<20 or >50 g/L), ALP (>URL), departmental exclusions	2587	0.0144 (0.0138–0.0151)	0.529	1.792 (1.769–1.814)	0.582
6. Only Ca, Alb and ALP exclusion criteria	Total calcium (<2.0 or >2.7 mmol/L), albumin (<20 or >50 g/L), ALP (>URL)	19,683	0.0141 (0.0138–0.0144)	0.184	1.818 (1.807–1.829)	0.041*
7. Only Ca and Alb exclusion criteria	Total calcium (<2.0 or >2.7 mmol/L), albumin (<20 or >50 g/L)	22,318	0.0136 (0.0133–0.0139)	0.020*	1.838 (1.828–1.848)	0.002**
8. No exclusions	None	22,906	0.0150 (0.0147–0.0153)	0.667	1.786 (1.776–1.796)	0.757

The mean total calcium and albumin concentrations of all seven subset groups were found to statistically significantly differ from that of the ACB group (Table 3). However, in each case, those groups with a higher mean total calcium than the ACB group also had higher mean albumin concentrations, while those with lower mean total calcium values had lower mean albumin values.

**Table 3.** Mean total calcium and albumin concentrations for each derivation group.

Derivation group	Total calcium		Albumin	
	Mean concentration (mmol/L)	*p*-value	Mean concentration (g/L)	*p*-value
1. All ACB exclusion criteria	2.310		35.82	
2. All ACB exclusion criteria bar Mg	2.346	<0.0001	37.52	<0.0001
3. All ACB exclusion criteria bar creatinine	2.292	0.0001	34.55	<0.0001
4. All ACB exclusion criteria bar ALT	2.292	<0.0001	34.68	<0.0001
5. All ACB exclusion criteria bar both creat and ALT	2.289	<0.0001	34.42	<0.0001
6. Only Ca, Alb and ALP exclusion criteria	2.351	<0.0001	37.72	<0.0001
7. Only Ca and Alb exclusion criteria	2.347	<0.0001	37.32	<0.0001
8. No exclusions	2.342	<0.0001	37.06	<0.0001

### Effect of equations on patient results

All eight adjusted calcium equations were applied to the validation cohort, containing 19,640 adults (10,937 female, 8699 male, four unknown; median age: 54.9 years [range 18.0–109.8]). Compared to the adjusted calcium results derived using the reference ACB criteria, no individual’s adjusted calcium value calculated using any of the other seven equations was found to be more than either 5% and 0.1 mmol/L different.

**Figure 1.**
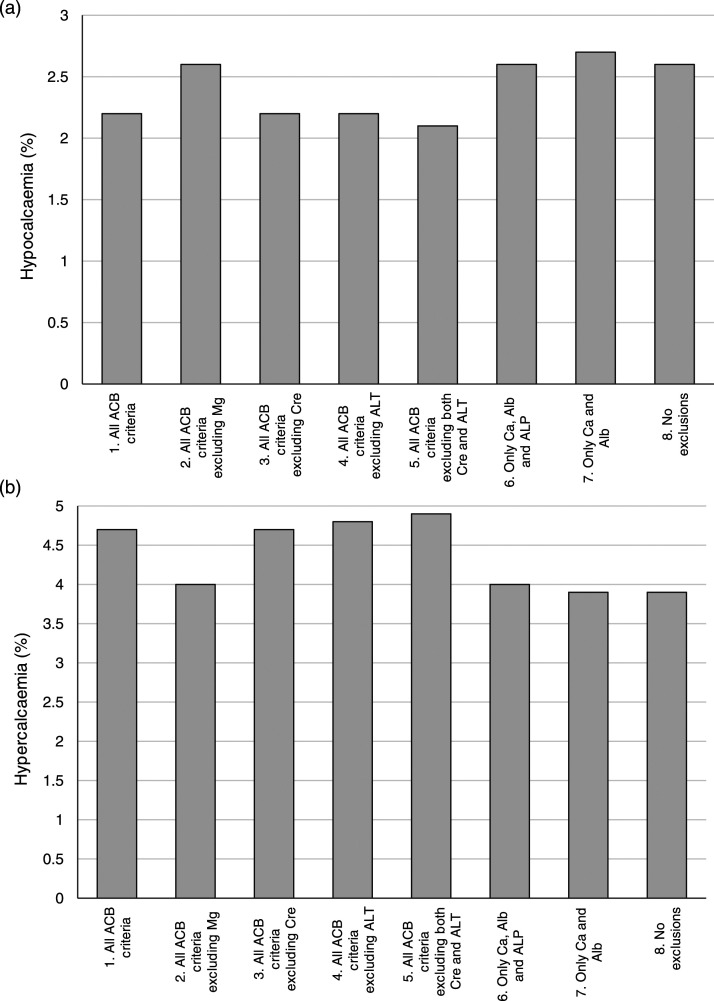
The effect of different exclusion criteria on the classification of calcium status. Hypocalcaemia: <2.2 mmol/L, normocalcaemia: 2.2–2.6 mmol/L, hypercalcaemia: >2.6 mmol/L.

The adjusted calcium concentrations calculated using each of the exclusion criteria equations were then classified using the Pathology Harmony reference range (2.2–2.6 mmol/L) (Figure 1). Again, the equations derived using all the ACB criteria except creatinine, except ALT, or without both creatinine and ALT (Groups 3, 4 and 5) classified almost identical numbers of patients as either hypocalcaemic or hypercalcaemic. Equation (7) (restricted only by the exclusion of extreme calcium and albumin values) disagreed the most with the calcium status classifications made by the reference ACB equation: the proportion of patients classed as hypocalcaemic was 0.51% higher, and the proportion classed as hypercalcaemic was 0.78% lower than the reference ACB equation.

The Cohen’s kappa score was used to assess the agreement between calcium status classification calculated using both the equation derived using all the ACB criteria (Equation (1)) and the equations where each subset of exclusions were applied (Table 3). None of the adjustment equations resulted in a kappa score below 0.90, demonstrating almost perfect agreement. In fact, a comparison between Equation 1, the reference ACB equation, and Equation 8, where no exclusion criteria were applied, produced a kappa score of 0.91 (95% CI 0.90–0.92) (Table 4).

**Table 4.** The agreement between the calcium status classification calculated using either the ACB calcium adjustment equation or one derived using a subset of exclusion criteria. Groups are numbered as in Table 2.

Agreement between calcium adjustment equations	Kappa (95% CI)
ACB versus Group 2	0.92 (0.91–0.93)
ACB versus Group 3	0.99 (0.98–0.99)
ACB versus Group 4	0.99 (0.98–0.99)
ACB versus Group 5	0.98 (0.98–0.99)
ACB versus Group 6	0.92 (0.91–0.93)
ACB versus Group 7	0.90 (0.89–0.91)
ACB versus Group 8	0.91 (0.90–0.92)
ACB versus Unadjusted Calcium	0.35 (0.33–0.37)

In contrast, classification using the unadjusted total calcium concentrations resulted in a markedly higher proportion of patients having hypocalcaemia (11.1% vs 2.2%, respectively) and a much smaller proportion were hypercalcaemic (2.0% vs 4.7%) compared with those classified using the ACB criteria adjustment equation. The disparity between the two designations of calcaemic status is reflected in the much lower kappa score of 0.35 (95% CI 0.33–0.37), demonstrating only fair agreement.

## Discussion

The ACB recommend the use of exclusion criteria when locally deriving calcium adjustment equations, with the aim of removing patients with possible calcium homoeostasis disorders.^8^ However, there is inconsistency in the literature regarding which exclusion criteria are used and the relative paucity of some of the recommended specialist measurements (such as PTH or vitamin D) makes meeting the ACB-recommended number of at least 1000 measurements difficult. Here, we have demonstrated that almost any combination of exclusion criteria, including when no exclusion criteria are used at all, does not make a clinically or statistically significant difference to the final equation.

Overall, the derivation subgroups with the numerically closest mean total calcium and albumin concentrations, slope and intercept values and calcium status classification to the reference ACB group were those that used all the ACB exclusion criteria except for the creatinine and/or ALT criteria (groups 3, 4 and 5). This suggests that the inclusion of patients with abnormal (or no) creatinine and/or ALT measurements does not significantly alter the performance of the adjustment equation. This contrasts with the mean albumin concentrations in the groups excluding no creatinine and/or ALT values, which were predictably lower than the ACB group. However, perhaps crucial to their adjustment equations, total calcium concentrations in these groups were lower too.

Historically, it is unclear why the specific exclusion criteria and associated concentration thresholds were chosen by Payne and subsequently the ACB, other than them being a pragmatic approach to identify patients with potential calcium homoeostasis disorders.^6,8^ Although not explicitly stated in these studies, patients with elevated creatinine were presumably excluded due to the prevalence of mineral and bone disorders in CKD patients, and the associated changes to calcium handling.^19^ Similarly, impaired liver function, indicated by elevated ALT, may result in altered production and structure of albumin, thus affecting the relationship between calcium and albumin.^20^ However, reasons why tests measuring other factors known to affect calcium binding, such as phosphate or globulins, are not included have not been stated.^21,22^ Instead, recommendations are made for exclusion of patients with malignancy, or attending oncology departments, even though this information is not always readily available when data is extracted from laboratory information systems for analysis.

It has been argued that the altered relationship of calcium and albumin in patients with impaired renal function makes the application of an adjustment equation derived from healthy patients inappropriate.^19^ Indeed, several studies have attempted to derive population-specific equations in patients with an eGFR of <60 mL/min/1.73 m^2^ or recommend abandoning the practice of adjusting total calcium concentrations entirely in this population.^12,23,24^ It is therefore perhaps surprising that in our study the inclusion of these patients in the derivation group makes little difference to the adjustment equation. One reason for this could be that a cut-off value for creatinine of >200 μmol/L already leads to the inclusion of most patients with renal impairment regardless of whether the exclusion criteria is applied or not. However, counting against this argument, Jassam et al.^25^ recently compared the calcium adjustment equations of 12 primary care sites derived using Payne’s criteria (excluding those with creatinine >200umol/L) with those where patients with an eGFR <60 mL/min/1.73 m^2^ were additionally excluded. They found the 12 respective pairs of equations to be identical. Reconciling these findings with this study would either suggest that even severe renal impairment does not have as large an influence on calcium adjustment as might be expected or that the degree of impairment needed is found in too few patients to materially influence an adjustment equation.

In contrast to patients with reduced renal function, there is very little literature that identifies liver patients as a group where adjusted calcium is not applicable. Certainly, in this study, inclusion of patients with elevated ALT did not result in a significantly different adjustment equation either.

Of note were the results we obtained from the group where no exclusion criteria were applied at all (Group 8). This equation did not significantly differ from the reference ACB equation in respect of slope or intercept values and, in fact, was the closest statistically (though not numerically) of all seven non-reference equations. It also classified the calcium status in patients (either hypo-, normo- or hypercalcaemia) very similarly to the reference ACB equation, showing almost perfect agreement using Cohen’s kappa statistic.

In contrast to this all-patient group, the group where the adjustment equation was most different to the reference ACB group was the one where only the extreme values of total calcium (<2.0 and >2.7 mmol/L) and/or albumin (<20 and >50 g/L) were excluded (Group 7). Despite containing only 587 (2.6%) fewer patients than the all-patient group (Group 8), it had the most different slope (*p*=0.002) and intercept values (0.002) compared to the reference ACB equation. This is particularly interesting as calcium and albumin exclusions of any sort are not made in many recent studies deriving adjustment equations, despite its recommendation in the ACB guidelines.^5–9,14–16,26,27^

Statistical significance in respect to equation slopes and intercepts, and whether any difference between equations could lead to clinically significant differences in the same patient, are two different things. Jassam et al. defined a clinically significant difference between calcium adjustment equation as being >5% or 0.1 mmol/L (whichever is greatest) but have also used the 4.6% minimal allowable error proposed by the biological variation model.^14,15,28^ When using the above criteria to determine clinical difference, we found that there was not a single person in the validation group of patients whose adjusted calcium result was clinically different when using the equation derived from Group 7 criteria compared to the reference ACB equation, nor did it translate to a marked change in calcium status classification, as the proportion of patients classed as hypocalcaemic rose by only 0.51% and the proportion of patients classed as hypercalcaemic fell by only 0.78%. This translated to a kappa score of 0.90 between Group 1 and Group 7 which, according to both Cohen’s and McHugh’s values for interpretation, still demonstrates almost perfect agreement between the two groups.^29^ Given that the Group 7 equation was furthest from the reference ACB one, it was not unexpected that there were no clinical differences in any of the other equation groups.

This study’s findings may have important clinical implications. For example, a concern highlighted earlier in this article is the inconsistency between laboratories in the exclusion criteria chosen when deriving local adjustment equations. Our study would suggest that this should not lead to a marked clinical difference between laboratories. Indeed, the data raises the question of whether exclusion criteria should be used at all, especially given that the current application of all recommended exclusion criteria is difficult, time-consuming and could potentially be irrelevant. If these findings were confirmed in subsequent studies, the omission of exclusion criteria could drastically simplify the process for deriving local adjustment equations.

Requiring fewer exclusions is also appealing when considering the derivation of age- and population-specific equations. For example, Jassam et al.^14^ found that the exclusion criteria dramatically limited the size of the younger derivation groups, despite analysing over 17,000 calcium and albumin concentrations from 12 months of data in three large teaching hospitals. This likely limits the ability to study age-specific equations generated from retrospective data and may partly explain the lack of agreement between ionized (or free) calcium and adjusted calcium in the younger age groups. The removal of the need for exclusion criteria, if applicable to paediatrics as well as adults, could therefore increase the size of the derivation groups and result in more dependable age-specific adjustment equations.

An important limitation to this study, which is common to many others, is that the clinical utility of the derived equations has not been compared to the true calcium status of the patient. Ionized calcium is generally treated as the gold standard measurement for the estimating calcium status, but its use as a reference method in a comparison study requires its simultaneous measurement with a total calcium and albumin measurement. This is seldom done in practice unless it is required as part of a prospective study and is therefore not a viable option for routine validation of an equation.

In conclusion, while the ACB’s recommended process for using patient data to derive an adjusted calcium equation includes numerous exclusion criteria, this study has shown that the individual or even complete omission of test exclusion criteria during the equation derivation process does not markedly change the final adjustment equation or how it classifies patients’ calcium status. If this robustness in any derived equation, irrespective of exclusion criteria used, were to be confirmed by other studies then it could potentially simplify the process of locally determining an adjustment equation.
